# Rural revitalization, carbon emissions, and infectious disease transmission in China: nonlinear dynamics, spatial spillovers, and moderating effect

**DOI:** 10.3389/fpubh.2026.1887653

**Published:** 2026-08-31

**Authors:** Quanyou Shang, Xiaoning Zheng, Yujue Wang

**Affiliations:** 1 Minzu University of China, Beijing, China; 2 Universiti Sains Malaysia, Penang, China

**Keywords:** China, CO_2_, infectious diseases, moderating effect, nonlinear dynamics, rural revitalization, spatial spillovers

## Abstract

Atmospheric pollution, particularly carbon dioxide (CO_2_) emissions driven by rapid economic development, poses significant threats to public health. In China, rural revitalization policies have transformed rural environments and socioeconomic structures, with implications for infectious disease transmission. This study examines the relationship between carbon emissions, rural revitalization, and infectious diseases using panel data from 31 provinces (2013–2023) within a spatial econometric framework. Results show that carbon emissions significantly increase disease transmission. Rural Revitalization exhibits a significant U-shaped non-linear effect, shifting from an initial negative impact that suppresses infection risks to a subsequent positive impact that elevates risks driven by enhanced population mobility and economic integration. Spatial Durbin Model reveals profound positive spatial spillover effects, demonstrating that both Carbon Emissions and Infectious Disease Transmission are highly interdependent across neighboring provinces. This indicates that a region’s epidemiological risk is heavily conditioned by the transboundary environmental pressures and developmental trajectories of its contiguous territories. These findings underscore that coordinating environmental and rural development policies through a transition toward stage-dependent, low-carbon rural planning and cross-regional collaborative governance is a vital prerequisite for mitigating public health expenditures and safeguarding long-term regional sustainability.

## 1 Introduction

Infectious diseases remain one of the most persistent public health challenges worldwide, and their transmission has become increasingly sensitive to environmental changes associated with climate change. Rising temperatures, altered precipitation patterns, and the increasing frequency of extreme weather events have reshaped ecological systems and created favorable conditions for pathogen survival, vector proliferation, and disease transmission. Among the various drivers of climate change, carbon dioxide (CO_2_) emissions occupy a central position because they not only contribute to global warming but also reflect emission-intensive patterns of industrialization and energy consumption. Although CO_2_ itself is not directly harmful to human health at ambient concentrations, it serves as an important proxy for a wide range of environmental stressors, including particulate matter (PM_2.5_), nitrogen oxides (NO_x_), and ground-level ozone, which have well-documented adverse effects on respiratory and immune systems. Furthermore, climate change induced by rising CO_2_ concentrations alters temperature, humidity, and ecological conditions, thereby influencing pathogen survival, vector distribution, and infectious disease transmission dynamics (1–3). Understanding how environmental pressures associated with carbon emissions influence infectious disease transmission has therefore become an increasingly important issue for environmental epidemiology and public health research.

**Figure 1:**
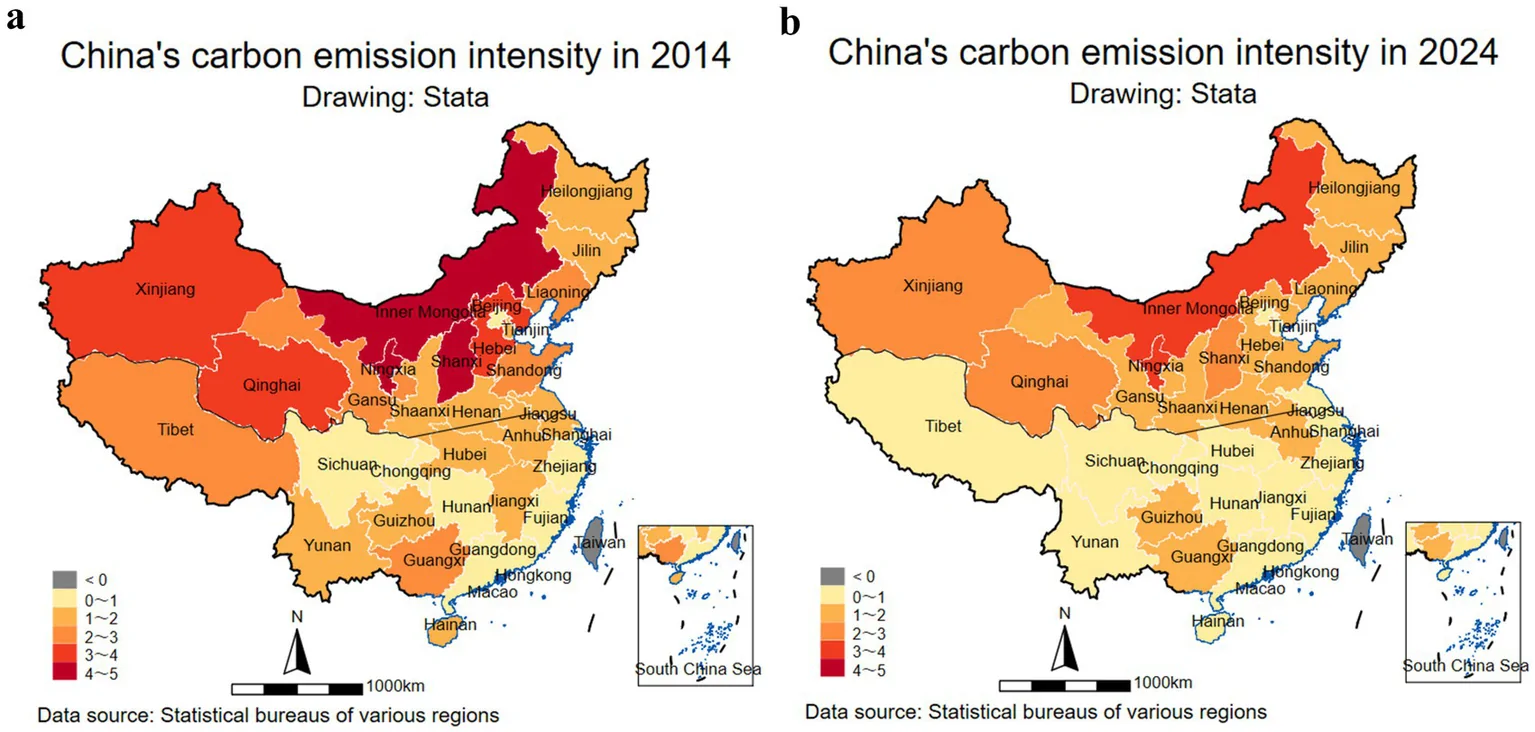
**(a)** Spatial distribution of carbon emission intensity in China, 2014. **(b)** Spatial distribution of carbon emission intensity in China, 2024.

China provides a particularly important context for examining these relationships because it simultaneously experiences rapid economic transformation, substantial environmental pressure, and profound institutional change. As the world’s largest carbon emitter, China contributes approximately 30% of global CO_2_ emissions while continuing to undergo rapid industrialization and urbanization (4). However, carbon emissions are distributed unevenly across provinces, reflecting considerable regional differences in industrial structure, energy consumption, and economic development. Such spatial heterogeneity implies that environmental risks and their associated health consequences are also likely to vary geographically rather than being uniformly distributed across the country as illustrated in Figures 1a,b.

At the same time, China has implemented the Rural Revitalization Strategy as a long-term national development policy to reduce urban–rural disparities and improve the sustainability of rural development (5). Since its implementation, substantial investments have been directed toward rural infrastructure, sanitation systems, ecological restoration, healthcare services, and public governance. Improvements in drinking water safety, waste management, transportation infrastructure, and primary healthcare have significantly enhanced rural living conditions, while ecological conservation and environmental management have become increasingly integrated into rural development policies. These institutional transformations suggest that rural revitalization may influence public health not only by improving healthcare accessibility but also by modifying the environmental conditions under which infectious diseases emerge and spread (6). Nevertheless, the interaction between rural revitalization and environmental pressure remains insufficiently understood.

**Figure 2:**
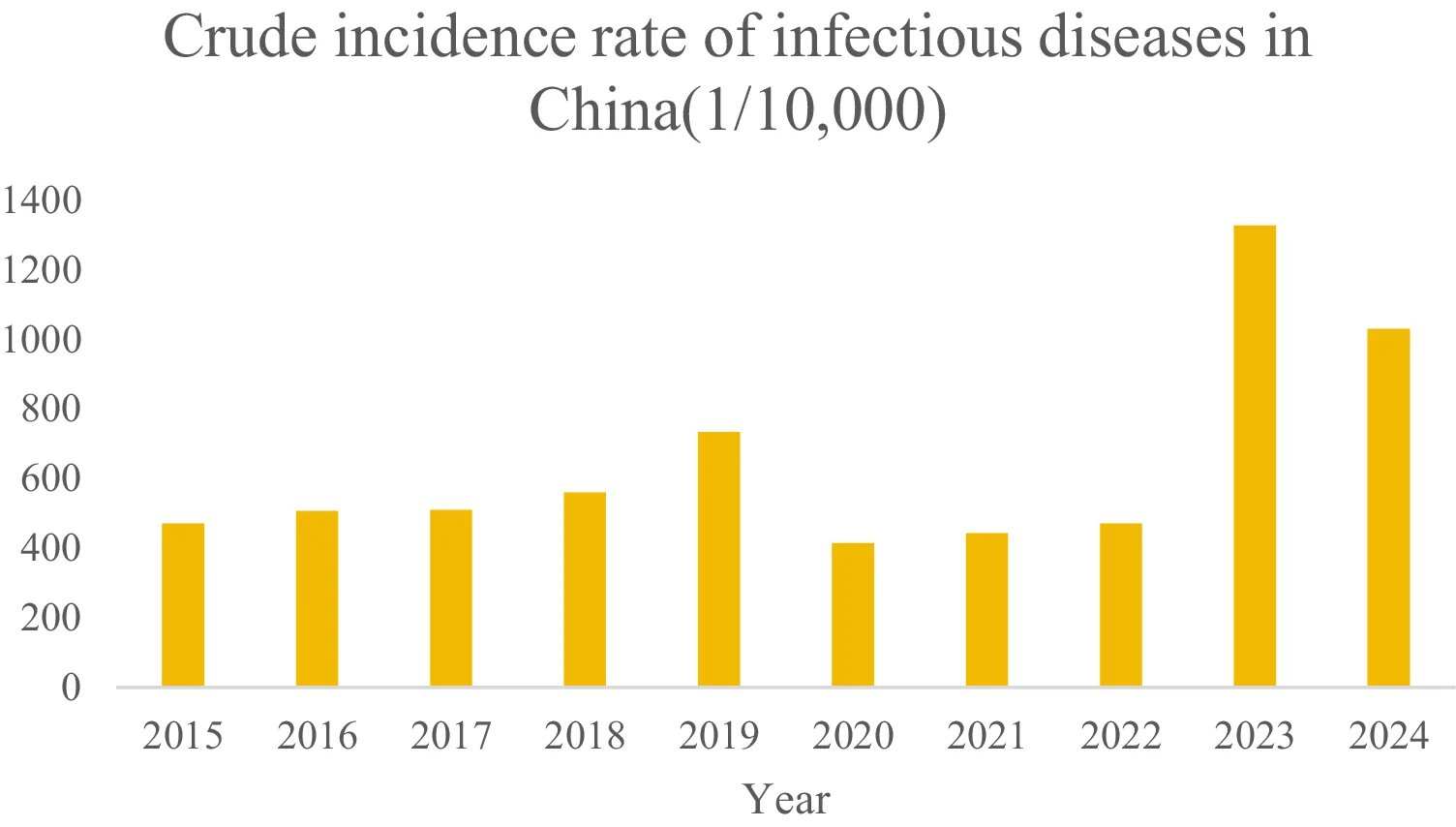
The crude incidence rate of infectious diseases in China. Source: National Bureau of Statistics of China, China Health Statistical Yearbook.

Meanwhile, infectious diseases continue to pose substantial public health challenges despite continuous improvements in healthcare systems. According to the China Health Statistical Yearbook, the incidence of Class B and Class C infectious diseases has exhibited considerable regional variation during the past decade, indicating that disease transmission is influenced by multiple environmental and institutional factors rather than by medical conditions alone in Figure 2. The continued increase in reported infectious disease incidence further highlights the importance of identifying the environmental and developmental factors that shape disease transmission under contemporary socioeconomic transformation.

Existing studies have generated important insights into the environmental and institutional determinants of infectious disease transmission. One major stream of research emphasizes the role of environmental conditions. A substantial body of evidence demonstrates that environmental pollution and climate change significantly influence infectious disease risks by altering ecological systems and pathogen survival conditions. Higher levels of air pollution have been consistently associated with increased infection risks (7), while Jang et al. (8) reported a strong positive relationship between air pollution and statutory infectious diseases in South Korea. Similarly, Ofremu et al. (9) suggested that greenhouse gas emissions are likely to intensify future climate-related health risks through changes in environmental conditions. Building upon the Health Production Theory proposed by Grossman, subsequent studies incorporated environmental externalities into health production models, arguing that pollution accelerates the depreciation of health capital (10, 11). More recent evidence further indicates that environmental pollution reduces life expectancy, increases disease burdens, and imposes substantial economic costs through pollution-related morbidity (12, 13). Collectively, these studies establish that environmental quality constitutes an important determinant of infectious disease transmission and broader population health.

A second strand of literature focuses on the institutional and socioeconomic determinants of infectious diseases. Previous research consistently shows that healthcare accessibility, sanitation infrastructure, education, and demographic characteristics substantially influence disease vulnerability. Higher-quality healthcare services are associated with lower infection risks among older populations (14), while improvements in medical services significantly reduce infection-related mortality among children (15). Aging populations are particularly vulnerable because declining immune function increases susceptibility to infectious diseases (16). Beyond healthcare systems, large-scale rural development initiatives also reshape local environmental and demographic conditions that influence epidemic dynamics (17). Early investments in sanitation infrastructure, public hygiene, and healthcare services generally reduce infectious disease transmission by improving environmental quality and strengthening community prevention capacity (18). However, continued rural modernization involving agricultural intensification, commercial development, land-use change, and increasing transportation connectivity may simultaneously introduce new exposure pathways by accelerating population mobility and environmental pressure (19). Consequently, existing evidence increasingly suggests that the relationship between rural development and public health may not be strictly linear but instead evolves dynamically throughout different stages of development (20).

Existing research has also increasingly recognized that infectious disease transmission is inherently spatial in nature. Unlike many chronic diseases, communicable diseases are capable of spreading rapidly across geographical boundaries through human mobility, environmental diffusion, and regional connectivity. Empirical evidence consistently demonstrates significant spatial clustering of infectious diseases. For example, Guo et al. (21) identified pronounced spatial aggregation of tuberculosis across western China, while Felipa (22) showed that regional disparities in healthcare accessibility substantially influence the effectiveness of infectious disease prevention. Other studies further indicate that health capital depreciation is strongly influenced by transboundary ecological disturbances and regional environmental interactions (23). Institutional interventions implemented in one region may also generate spillover effects on neighboring areas through interconnected healthcare systems, transportation networks, and population migration (24). These findings collectively suggest that infectious disease transmission should be regarded as a spatially interconnected process rather than an isolated local phenomenon. Ignoring spatial dependence may therefore lead to biased estimates and incomplete interpretations of the environmental determinants of disease transmission.

Although previous studies have substantially advanced understanding of environmental health and infectious disease epidemiology, several important limitations remain. First, most existing studies examine the independent health effects of environmental pollution or climate change, whereas considerably less attention has been devoted to understanding how institutional transformation influences the relationship between environmental pressure and infectious disease transmission. In particular, rural revitalization has primarily been investigated from the perspectives of economic development, poverty reduction, or agricultural modernization, with relatively limited empirical evidence regarding its role in shaping environmental health outcomes.

Second, previous research provides inconsistent conclusions regarding the health consequences of rural development. One group of studies argues that improvements in sanitation infrastructure, healthcare accessibility, and public services generated by rural development substantially reduce infectious disease risks (18, 25, 26). In contrast, other studies suggest that rapid rural transformation may increase disease transmission by accelerating transportation connectivity, population mobility, and demographic concentration (27–31). Rather than representing contradictory evidence, these competing findings may indicate that the health effects of rural development evolve across different stages of development. However, few studies have explicitly examined whether the relationship between rural revitalization and infectious disease transmission follows a nonlinear trajectory, leaving the dynamic nature of rural transformation insufficiently understood.

Third, despite growing recognition of regional interdependence, the interaction between environmental pollution and infectious disease transmission has largely been examined using conventional panel regression models that assume regional independence. Such approaches overlook the atmospheric diffusion of environmental pollutants, ecological continuity across neighboring regions, and the spatial transmission of infectious diseases through transportation networks and population movement. Consequently, the regional spillover effects generated by both environmental degradation and institutional development remain inadequately captured.

Although infectious diseases are commonly classified into administrative categories for surveillance and public health management, relatively few empirical studies have evaluated whether environmental and institutional effects remain consistent across diseases with different epidemiological characteristics. Most existing analyses rely on aggregated disease indicators without examining whether the observed relationships are robust across representative diseases with distinct transmission pathways. Consequently, it remains unclear whether previously identified environmental-health relationships reflect genuine epidemiological mechanisms or simply arise from aggregation across heterogeneous infectious diseases.

These limitations indicate the need for a more integrated analytical framework capable of simultaneously incorporating environmental pressure, institutional transformation, nonlinear development processes, and spatial interactions. Such a framework is particularly important for understanding infectious disease transmission during China’s ongoing rural transformation, where environmental sustainability and rural modernization are being pursued simultaneously under the Rural Revitalization Strategy.

From a theoretical perspective, this study extends the Health Production Theory by incorporating both environmental externalities and institutional governance into a unified analytical framework. Traditional health production models conceptualize health as a form of capital that is influenced by individual investment, healthcare services, and environmental conditions (Grossman) (10, 11). Building upon this perspective, the present study argues that rural revitalization influences the relationship between carbon emissions and infectious disease transmission through two complementary pathways.

The first pathway operates through environmental improvement. Investments in rural infrastructure, ecological restoration, pollution control, sanitation systems, and environmental governance improve local environmental quality and reduce exposure to climate-related environmental risks associated with carbon emissions. These improvements create less favorable ecological conditions for pathogen survival and interrupt important transmission pathways for environmentally sensitive infectious diseases.

The second pathway operates through institutional and social capacity. Rural revitalization strengthens primary healthcare systems, expands healthcare accessibility, improves public health literacy, and enhances local governance capacity. These institutional improvements increase communities’ ability to prevent, detect, and respond to infectious disease outbreaks, thereby reducing population vulnerability to environmental health risks. Rather than functioning independently, these environmental and institutional mechanisms jointly determine how environmental pressure is translated into infectious disease transmission.

Based on this theoretical framework, this study investigates the relationships among carbon emissions, rural revitalization, and the transmission of Class B and Class C infectious diseases across 31 Chinese provinces from 2013 to 2023. Compared with aggregate indicators such as mortality or life expectancy, infectious disease incidence provides a more direct measure of disease transmission dynamics and therefore offers a more appropriate basis for evaluating environmental health risks. Furthermore, by incorporating spatial econometric models and disease-specific heterogeneity analyses, this study seeks to capture the complex interactions among environmental change, institutional transformation, and regional disease diffusion.

The study addresses three interrelated research questions. First, how do carbon emissions and rural revitalization independently influence infectious disease transmission, and does this relationship exhibit significant nonlinear characteristics? Second, does rural revitalization mitigate the adverse health effects associated with carbon emissions by strengthening environmental governance and institutional capacity? Third, do carbon emissions and rural revitalization generate significant spatial spillover effects that extend beyond provincial administrative boundaries, and are these relationships robust across representative infectious diseases with different epidemiological characteristics?

To answer these questions, this study makes three principal contributions to the existing literature. First, it integrates rural revitalization into the environmental-health framework by examining its moderating role in the relationship between carbon emissions and infectious disease transmission, thereby extending previous research that has largely considered environmental and institutional factors separately. Second, it demonstrates that the health consequences of rural revitalization are characterized by a nonlinear developmental process rather than a simple linear relationship, providing a more comprehensive understanding of rural transformation and public health. Third, by combining spatial econometric analysis with disease-specific heterogeneity tests, this study offers more robust evidence regarding the environmental and institutional determinants of infectious disease transmission while explicitly accounting for regional interdependence and differences across representative diseases.

The remainder of this paper is organized as follows. Section 2 describes the data, variables, and empirical methodology. Section 3 reports the empirical results, including the baseline estimations, mechanism analysis, spatial effects, and disease-specific heterogeneity analyses. Section 4 discusses the findings and their theoretical and policy implications. Finally, Section 5 concludes the study.

## 2 Methodology

### 2.1 Hypothesis formulation

Building upon the above literature, this study develops a set of testable hypotheses to examine the relationship between carbon emissions, rural revitalization, and infectious disease transmission within a unified analytical framework.

#### 2.1.1 Carbon emissions and infectious disease transmission

Existing studies have established that environmental degradation plays a significant role in shaping public health outcomes. In particular, carbon emissions contribute to climate change, air pollution, and ecological disruption, all of which influence the transmission environment of infectious diseases. Rising temperatures and deteriorating air quality may enhance pathogen survival, increase human exposure, and intensify the spread of infectious diseases. At the same time, the health production framework suggests that environmental stressors accelerate the depreciation of health capital, thereby increasing disease vulnerability. However, the relationship between carbon emissions and health outcomes may not be strictly linear. In the early stages of development, increases in carbon emissions are often accompanied by environmental degradation and rising health risks. As economic development progresses, improvements in environmental regulation, technological innovation, and public health systems may mitigate these adverse effects. This dynamic is consistent with a nonlinear relationship between environmental pressure and health outcomes.

*H1:* Carbon emissions positively affect infectious diseases.

#### 2.1.2 Rural revitalization and infectious disease transmission

Existing studies have established that comprehensive rural development and institutional transformations play a significant role in shaping public health outcomes. In particular, rural revitalization contributes to infrastructure upgrading, ecological governance, and economic integration, all of which influence the transmission environment of infectious diseases. Enhanced sanitation facilities and modernized waste management may suppress pathogen survival, decrease human exposure, and contain the spread of infectious diseases. At the same time, the health production framework suggests that expanding public services and rural medical accessibility bolsters health capital accumulation, thereby decreasing disease vulnerability. However, the relationship between rural revitalization and health outcomes may not be strictly linear. In the early stages of implementation, comprehensive environmental cleanup and basic sanitation improvements serve as primary protective barriers against epidemics. As rural development progresses into advanced stages, heightened commercial connectivity, industrial concentration, and intensified cross-regional population mobility may conversely expand pathogen transmission channels. This dynamic is consistent with a non-linear U-shaped relationship between rural institutional progress and health outcomes.

*H2:* Rural revitalization has a U-shaped non-linear effect on infectious diseases.

#### 2.1.3 Spatial spillover effects

Infectious diseases are inherently spatial in nature, as their transmission depends on population mobility, geographic proximity, and regional interactions. Similarly, carbon emissions and environmental pollution often exhibit spatial spillover effects due to atmospheric diffusion and economic linkages across regions. As a result, the impact of carbon emissions in one region may extend beyond its boundaries, affecting neighboring areas. Ignoring spatial dependence may lead to biased estimation results and an incomplete understanding of the underlying mechanisms. Incorporating spatial interactions allows for a more accurate assessment of both environmental externalities and disease transmission dynamics.

*H3:* Carbon emissions and infectious disease transmission exhibit significant spatial spillover effects.

#### 2.1.4 Moderating role of rural revitalization

While carbon emissions shape the environmental conditions of disease transmission, institutional and socio-economic factors determine how these environmental risks translate into actual health outcomes. Rural revitalization, as a comprehensive development strategy, may influence this relationship by improving infrastructure, public services, and governance capacity. On the one hand, improved environmental management and infrastructure investment can reduce population exposure to pollution-related risks. On the other hand, enhanced access to healthcare services, education, and public health resources can strengthen disease prevention and reduce vulnerability. These effects suggest that rural revitalization does not directly eliminate environmental pressures but instead alters the sensitivity of health outcomes to such pressures.

*H4:* Rural revitalization moderates the relationship between carbon emissions and infectious disease transmission, weakening the adverse health effects associated with carbon emissions.

#### 2.1.5 Mechanism analysis: environmental and social pathways

To further clarify the underlying mechanisms, this study considers two primary channels through which rural revitalization operates. The first is an environmental pathway. Improvements in environmental governance and pollution control can reduce the intensity of environmental degradation, thereby weakening the impact of carbon emissions on disease transmission. The second is a social pathway. Enhancements in healthcare accessibility, public health investment, and education can improve adaptive capacity and reduce the likelihood of infection. These channels suggest that the moderating effect of rural revitalization is not purely statistical but operates through observable structural changes in environmental conditions and social systems.

*H5:* Rural revitalization influences infectious disease transmission through environmental and social pathways.

### 2.2 Model specification

Building on the hypotheses developed in the previous section, this study proceeds to construct an empirical framework to examine the relationship between carbon emissions, rural revitalization, and infectious disease transmission. The hypotheses suggest that carbon emissions affect health outcomes in a potentially nonlinear manner, while rural revitalization moderates this relationship through institutional and socio-economic channels. An important feature of infectious disease transmission and environmental pollution is their strong spatial dependence. Infectious diseases are not confined to administrative boundaries; their spread is influenced by population mobility, geographic proximity, and interregional interactions. Similarly, carbon emissions and related environmental pollutants can diffuse across regions through atmospheric circulation and economic linkages. As a result, the health effects of carbon emissions in one region may extend to neighboring areas.

The theoretical framework of this study is based on the health production function proposed by Grossman. In econometric terms, the model is formulated in Equation 1 as follows:

h=F(Y,S,V) (1)

Here Y represents the per capita vector of economic status indicators S and V represent per capita social and lifestyle factor indicators and environmental factor indicators, respectively. The above health production function is refined into a concrete form using the differential method in Equation 2 as follows.

$$\text{diseas}e_{\mathrm{it}}=\sum \alpha_{i}(y_{i})+\sum \beta_{j}(s_{j})+\sum \gamma_{k}(\nu_{k})$$ (2)

The transmission of infectious diseases is fundamentally a spatial process governed by geographical proximity and regional connectivity. Pathogen diffusion does not respect administrative boundaries, and cross regional population mobility further accelerates transboundary health shocks. It is necessary to adopt a spatial econometric approach that explicitly incorporates spatial interactions into the empirical analysis. In light of these considerations, this study employs a spatial econometric model to capture both the direct effects of carbon emissions and the indirect effects transmitted across regions. This approach allows for a more comprehensive assessment of the environmental–health relationship within a spatially interconnected system. The model integrates the effects of rural revitalization policies and regional CO_2_ emissions, and empirically assesses their spatial impacts on the incidence of Category B and C infectious diseases. Accordingly, spatial econometric models are developed in Equations 3 and 4 as follows:

$$\begin{array}{l} \text{diseas}e_{\mathrm{it}}==\rho \sum_{j=1}^{n}W_{\mathrm{ij}}\text{diseas}e_{\mathrm{jt}}+\alpha 1{\mathrm{RCE}}_{\mathrm{it}}+\beta 1\sum_{j=1}^{n}W_{\mathrm{ij}}{\mathrm{RCE}}_{\mathrm{it}}\\ +\alpha 2{\mathrm{RR}}_{\mathrm{it}}+\beta 2\sum_{j=1}^{n}W_{\mathrm{ij}}RR_{\mathrm{it}}+\alpha 3{\left(\mathrm{RCE}*\mathrm{RR}\right)}_{\mathrm{it}}\\ +\beta 3\sum_{j=1}^{n}W_{\mathrm{ij}}{\left(\mathrm{RCE}*\mathrm{RR}\right)}_{\mathrm{it}}+\omega X_{\mathrm{jt}}+\tau \sum_{j=1}^{n}W_{\mathrm{ij}}X_{\mathrm{jt}}+\eta_{i}+\delta_{t}+\mu_{\mathrm{it}} \end{array}$$ (3)

$$\mu_{\mathrm{it}}=\lambda \sum_{j=1}^{n}W_{\mathrm{ij}}\mu_{\mathrm{jt}}+\nu_{\mathrm{it}}$$ (4)

Where disease denotes the incidence rate of infectious diseases of category B and C, and W_ij_ is the spatial weight matrix. Drawing on the method of Zhang and Zhu (32), we construct the inverse geographic distance spatial weight matrix, which is calculated as follows: W_ij_ = 1/D_ij_, where D_ij_ denotes the distance between the latitude and longitude geographic locations of province i and province j. The closer the distance between the two, the stronger the impact, and vice versa, the weaker the impact. RCE denotes the regional CO2 emissions, RR denotes the moderating variable of rural revitalization policy, and X_it_ is the control variable in the model. The control variables in this paper are Marketization degree index (MDI), Investment efficiency (IE), labor compensation (LC) and Urban and Rural Structure (URS), with i, j representing provincial and municipal cross-section units, ηi and δt represent individual and time effects, respectively, and μit is a random disturbance term. For the specific choice of model, this paper will screen the corresponding model parameters based on LM test, LR test, Wald test and other methods.

### 2.3 Econometric methods

Unlike standard regression models, this paper uses provincial spatial weight matrices, which means that variables in one province can influence neighboring provinces as well as their own province. To account for this, the paper calculates both direct and indirect effects of the independent variables on the dependent variables in the spatial model. This calculation follows the approach of Pace and LeSage (33), based on their interpretation of spatial model parameters in the bias matrix. The results for the direct and indirect effects of the independent variables on the dependent variables, as formulated in Equation 5, are as follows.

$$\begin{array}{l} \left[\frac{\partial \text{disease}}{\partial X_{1}}\cdots \frac{\partial \text{disease}}{\partial X_{n}}]=[\begin{array}{c} \frac{\partial \text{diseas}e_{1}}{\partial X_{1}}\cdots \frac{\partial \text{diseas}e_{1}}{\partial X_{n}}\\ \ldots \\ \frac{\partial \text{diseas}e_{n}}{\partial X_{1}}\cdots \frac{\partial \text{diseas}e_{n}}{\partial X_{n}} \end{array}\right]\\ ={\left(I-\mathrm{\rho W}\right)}^{-1}(\mathrm{\alpha I}+\mathrm{\beta W}) \end{array}$$ (5)

Whether rural revitalization policies and the spread of infectious diseases are spatially dependent in general was tested using the Global Moran I index, as expressed in Equation 6:

$${\text{Moran}}^{’}s\;I=\frac{n\sum_{i=1}^{n}\sum_{j=1}^{n}w_{\mathrm{ij}}(x_{i}-\bar{x})(x_{j}-\bar{x})}{\sum_{i}^{n}\sum_{j=1}^{n}w_{\mathrm{ij}}\sum_{i=1}^{n}{\left(x_{i}-\bar{x}\right)}^{2}}$$ (6)

where x_i_ and x_j_ denote the indicators for spatial units i and j, respectively, *n* denotes the total number of spatial units, and w_ij_ denotes the spatial weight matrix. Thirty-one provinces (cities) in China were analyzed, with *n* equal to 31.

While the global Moran’s I index highlights the overall patterns of Class B infectious disease transmission, it does not capture the specific characteristics of individual provinces. In contrast, the local Moran’s I index can reveal individual variations and provide detailed insights into each province. The local Moran’s I index for each province is calculated as follows:

$$\text{Local}\;{\text{Moran}}^{’}s\;I_{i}=\frac{n(y_{i}-\bar{y})\sum_{j=1}^{n}d_{\mathrm{ij}}(y_{j}-\bar{y})}{\sum_{j=1}^{n}d_{\mathrm{ij}}{\left(y_{j}-\bar{y}\right)}^{2}}$$ (7)

In Equation 7, each symbol denotes the same meaning as the global Moran I index. Calculate the local Moran I index equation for each province for 2013–2023.

Prior to the spatial econometric analysis, we tested the existence of spatial autocorrelation and verified the plausibility of the chosen spatial model. The basic panel model is shown in Equation 8:

$$y_{\mathrm{it}}=\alpha_{0}+\alpha_{1}X_{\mathrm{it}}+\alpha_{2}Z_{\mathrm{it}}+\varepsilon_{\mathrm{it}}$$ (8)

Where I and t denote the I th province in year t, respectively, y denotes the composite index in the transmission of B infectious diseases, X represents the core explanatory variable of rural revitalization policy and the intensity of regional CO2 emissions and its interaction term, and Z denotes the set of other control variables affecting the transmission of B infectious diseases, which in this case refers to degree economic development, marketization, foreign direct investment, and urbanization.

To empirically validate the pathways of Ecological Buffering Capacity and Comprehensive Health Literacy, we construct a mediation model. Expanding upon the baseline spatial Durbin framework, the mediation equations are specified in Equations 9 and 10 as follows:

$$M_{\mathrm{it}}=\theta_{0}+\theta_{1}{\mathrm{RR}}_{\mathrm{it}}+\phi X_{\mathrm{it}}+\eta_{i}+\delta_{t}+\mu_{\mathrm{it}}$$ (9)

$$\begin{array}{l} {\text{Disease}}_{\mathrm{it}}=\rho \sum_{j=1}^{n}W_{\mathrm{ij}}{\text{disease}}_{\mathrm{jt}}+\alpha_{1}{\mathrm{RR}}_{\mathrm{it}}\\ +\alpha_{2}\sum_{j=1}^{n}W_{\mathrm{ij}}{\mathrm{RR}}_{\mathrm{it}}+\gamma_{1}M_{\mathrm{it}}+\omega X_{\mathrm{it}}+\eta_{i}+\delta_{t} \end{array}$$ (10)

Where $M_{\mathrm{it}}$ represents the respective mediating variables (Ecological Buffering Capacity and Comprehensive Health Literacy). The first equation employs a fixed-effects panel model to estimate coefficient $\theta_{1}$ , which isolates the influence of rural revitalization on the mediating pathway. The second equation incorporates the mediator into the baseline spatial Durbin model, where $\gamma_{1}$ estimates the subsequent impact of the mediator on infectious disease transmission. If both $\theta_{1}$ and $\gamma_{1}$ are statistically significant, the mediating effect is confirmed.

### 2.4 Variable and data

This section defines the variables and describes the associated data. Provincial-level data in China are extracted and used to test the effects of regional rural revitalization policy implementation and regional CO2 emissions on regional infectious disease transmission. Meanwhile, this paper establishes several indicators to measure regional CO2 emissions and rural revitalization policies based on previous studies.

#### 2.4.1 Dependent variable: the infectious quantity of class B infectious diseases (CBID) and class C infectious diseases (CCID)

Regarding infectious diseases, China categorizes statutory infectious diseases into three classes—Category A, B, and C—based on their severity, transmissibility, and societal impact. In this paper, the number of each type of infectious disease is divided by the population of the region as the regional incidence rate to measure the extent of the spread of every kind of contagious disease and the percentage of each type of primary infectious disease during the decade 2013–2023 is shown in Table 1.

**Table 1:** Percentage of incidence of infectious diseases in China, 2013–2023.

Name of the infectious disease	%	Source
Category A infectious disease	0.05	Zheng et al. (37)
Category B infectious disease	32.17	Zhu et al. (38)
Viral hepatitis	15.20	World Health Organization (39)
Syphilis	5.78	Liao et al. (40)
TB	4.54	Liao et al. (40)
Other category B infectious diseases	6.65	Liao et al. (40)
Category C infectious diseases	67.78	Wang et al. (41)
Influenza	28.26	Liang et al. (42)
Hand, foot, and mouth disease (HFMD), caused by several intestinal viruses, usually affects young children	27.76	Wang et al. (41)
Other infectious diarrheal diseases	8.42	Li et al. (43)
Other category C infectious diseases	3.34	Miramontes Carballada and Balsa-Barreiro (44)
Total	100	Miramontes Carballada and Balsa-Barreiro (44)

#### 2.4.2 Independent variable: rural revitalization

The Independent variable in the investigation refers to the holistic measure of rural revitalization. Given the performance of various dimensions of Rural revitalization, referring to Xu and Wang (34), this paper comprehensively considers the five dimensions of industrial prosperity, ecological livability, civilized rural culture, effective governance, and affluent life, as well as data availability, and finally adopts 18 indicators as sub-dimensions to construct the rural revitalization index system, and adopts the entropy method of objective weighting to measure, and the comprehensive of rural revitalization development is shown in Table 2.

**Table 2:** Composite of rural revitalization (RR).

Domain	Elements
Flourishing industry	Primary sector output per capita. Grain production. Gross power of agricultural machinery. Effective irrigated area.
Ecological livability	Forest cover Non-hazardous domestic waste disposal rate Number of public toilets Number of rural doctors and health workers Integrated production capacity for water supply
Rural style civilization	Local financial expenditure on education. Illiterate population/population aged 15 and over. Integrated population coverage of television programs.
Efficient governance	Per capita disposable income of rural residents/per capita disposable income of urban residents. Per capita consumption expenditure of rural residents/per capita consumption expenditure of urban residents. Rural population.
Thriving livelihood	Disposable income per rural resident. Rural consumption expenditure on food. Rural self-employment quorum.

The selection of indicators for rural revitalization is grounded in the multidimensional nature of rural development. Disposable income and the urban–rural income ratio are included as proxies for health capital investment capacity and social equity. From a theoretical perspective, higher disposable income enables households to shift from basic subsistence to health-promoting consumption, thereby enhancing nutritional immunity and reducing susceptibility to pathogens. The narrowing of the urban–rural income gap reflects the equalization of public health services, which is critical for mitigating the spatial diffusion of infectious diseases. Food expenditure serves as an indicator of the consumption structure; as it reflects the transition toward higher-quality diets, it strengthens the biological resilience of rural populations against environmental stressors.

#### 2.4.3 Independent variable: regional carbon emissions

Referring to the study of Cong et al. (35), this paper takes into account all direct emissions within activities within the province, and other indirect emissions generated outside the jurisdiction of the province but not within the jurisdiction of the province and other indirect emissions generated outside the jurisdiction of the province but not included in the above two scopes. The results of this study are summarized in the Table 3, as well as the data availability, and ultimately adopts seven indicators as sub-indicators to construct the regional carbon emissions, and adopts the entropy method with objective weighting. Entropy method with objective weighting to measure the regional carbon emissions.

**Table 3:** Measurement of regional carbon emissions (RCE).

Domain	Element
All direct emissions within the jurisdiction of each province	Transport and construction Industrial process. Agroforestry and land-use change. Waste disposal
Indirect energy-related emissions outside the jurisdiction of the provinces	Purchased electricity. Heating and cooling
Other indirect emissions	Greenhouse gas emissions from production, transport, use, and waste disposal outside jurisdictions

To address the complexity of environmental health risks, the regional carbon emission index accounts for the heterogeneous impacts of sectoral sources. Industrial emissions often represent the concentration of secondary industries, which are frequently accompanied by co-pollutants that impair respiratory defenses and exacerbate the severity of infectious outbreaks. Agricultural carbon emissions are closely linked to land-use changes and livestock intensity, which influence zoonotic disease reservoirs and spillover risks. Transport-related emissions serve as a proxy for human mobility and regional connectivity; high transport intensity facilitates the rapid geographical transmission of infectious agents. By integrating these dimensions, the index captures the comprehensive ecological footprint that modulates the public health landscape.

#### 2.4.4 Mediating variables

To explore the internal transmission mechanisms, this study defines two critical mediators: Ecological Buffering Capacity (EBC) and Comprehensive Health Literacy (CHL). The former is measured by PM2.5 concentrations, reflecting the ability of rural environmental governance to absorb and mitigate the negative externalities of carbon emissions. A lower concentration signifies a robust ecological buffer that protects public health from atmospheric hazards. The latter, Comprehensive Health Literacy, is proxied by the density of students. This indicator represents the community’s potential for health capital accumulation and behavioral adaptation, as schools serve as primary hubs for disseminating hygiene knowledge and preventive protocols.

#### 2.4.5 Control variables

According to China’s provincial marketization index Wang and Tang (36), which includes the relationship between the government and the market, the development of the non-state economy, the degree of development of the product market, the degree of development of the factor market, the development of market intermediary organizations, and the rule of law environment, and consists of 18 primary indices adopt the entropy method with objective weighting to measure the marketization. Objective weighting to measure the marketization degree.

With regard to investment efficiency (IE), labor compensation (LC) and urban–rural structure (URS), the spread of infectious diseases is affected by investment efficiency (IE), labor compensation (LC) and urban–rural structure (URS), the spread of infectious diseases is affected by multiple factors, including economic, social and environmental factors. On the economic level, investment efficiency reflects the status of technology utilization and the degree of economic innovation and development in the process of economic development. At the level of social development, the gap between urban and rural structure is an important feature of China’s social economy. Finally, because the consumption gap is more important than the income and wealth gap, the weight of workers’ salary is used to evaluate the realization of environmental development factors.

In this paper, panel data of 31 provinces in China (excluding Hong Kong, Macao and Taiwan) from 2013 to 2023 are selected. The data used are from China Statistical Yearbook, China Health Statistics Yearbook, China Population and Employment Statistics Yearbook, China Labor Statistics Yearbook, National Economic and Social Development Statistics Bulletin, provincial and municipal Statistical Yearbooks, the National Bureau of Statistics, the database of the National Research Network, and the database of Guotai Junan.

Building upon these data sources and indicator systems, the specific classifications, symbols, operational definitions, and corresponding measurement methodologies for all variables utilized in the empirical analysis are synthesized in Table 4.

**Table 4:** Description and measurement of variables.

Variable type	Variable symbol	Variable description	Measurement method
Dependent	CBID	The infectious quantity of Class B infectious diseases	The number of infectious diseases/the population of the region
Dependent	CCID	The infectious quantity of Class C infectious diseases	The number of infectious diseases/the population of the region
Independent	RCE	Regional carbon emissions	Regional carbon emissions index system
Independent	RR	Rural revitalization level	Rural revitalization index system
Control	MDI	Marketization degree index	Marketization degree index system
Control	IE	Investment efficiency	The ratio of total fixed asset investment to regional GDP
Control	URS	Urban–rural structure	The proportion of urban population to the total population
Control	LC	Labor compensation	The weight of workers’ salary
Mediating	EBC	Ecological Buffering Capacity	PM2.5 concentrations
Mediating	CHL	Comprehensive Health Literacy	The density of students in the area

## 3 Results

### 3.1 Baseline regression results

The variance inflation factor (VIF) is used to test the data for the presence of multicollinearity before conducting the empirical analysis. As can be seen in Table 5, the maximum value of variance inflation factor is close to 7 and less than 10. therefore, multicollinearity can be ignored in the empirical analysis of panel data.

**Table 5:** Multicollinearity test.

Variable	VIF	1/VIF
RCE	2.07	0.4829
RR	2.05	0.4884
URS	5.99	0.1669
MDI	1.90	0.5255
LC	2.32	0.4308
IE	3.85	0.2595

The results of the non-spatial panel parameter estimates and spatial autocorrelation tests are shown in Table 6.

**Table 6:** Estimation results of panel data models without spatial interaction effects.

Type of disease	Category B infectious disease				Category C infectious diseases			
Variable	Pooled OLS	Spatial effects	Time-period fixed effects	Spatial and time-period fixed effects	Pooled OLS	Spatial effects	Time-period fixed effects	Spatial and time-period fixed effects
RCE	11.453*** (7.732)	5.487** (2.587)	15.543** (4.175)	4.546** (2.114)	5.507 (1.533)	2.831* (2.112)	5.742 (1.704)	3.886** (2.458)
RR	−0.432* (−1.885)	−0.438*** (−7.578)	−0.121*** (−6.550)	−0.146*** (−6.672)	−0.651** (−3.243)	−0.191 (−1.559)	−1.427*** (−4.543)	−0.386** (−3.447)
RR^2^	0.154*** (8.453)	0.045*** (3.245)	0.042*** (7.511)	0.025** (3.512)	0.035*** (4.418)	0.005 (0.957)	0.088*** (6.912)	0.009 (1.572)
RR*RCE	−1.161*** (−4.271)	0.759 (0.324)	−1.747*** (−4.445)	0.088 (0.444)	−0.120 (−0.389)	0.296** (2.057)	−0.480 (−1.636)	0.312** (2.160)
URS	−0.097 (−0.867)	0.042 (0.657)	0.124 (0.812)	0.056 (0.513)	−0.566** (−6.891)	−0.168** (−3.320)	0.106 (0.597)	−0.450*** (−2.758)
MDI	−3.453*** (−6.441)	−0.875*** (−2.824)	−3.452** (−5.504)	−0.557 (−1.498)	−1.214*** (−3.707)	−0.123 (−0.502)	−1.543*** (−4.203)	−0.092 (−0.242)
LC	−0.021 (−0.058)	0.891*** (2.757)	−1.075** (−2.168)	1.108** (3.271)	−1.493*** (−3.196)	0.514 (1.470)	−2.012*** (−4.373)	0.567 (1.592)
IE	−0.033 (−0.161)	0.470** (5.291)	−0.188 (−0.921)	0.495*** (5.519)	0.200** (2.364)	0.171*** (3.362)	0.034 (0.425)	0.167*** (3.295)
σ2	0.688	0.028	0.221	0.027	0.281	0.030	0.236	0.109
R2	0.682	0.157	0.706	0.169	0.627	0.088	0.686	0.029
LogL	−318,856	176,392	−305,001	181.556	−360,335	158,213	−320.008	165.297
LMlag	32.654***	0.463	31.254***	3.175*	5.555**	1.447	12.211***	5.750**
LMerror	41.547***	0.169	40.754***	3.436*	4.956*	0.927	3.238*	5.066**
LR test Spatial fixed effects Time-period fixed effects	LR=879.135, *p* = 0.001 LR=11.453, *p*=0.068				LR=863.463, *p* = 0.001 LR=13.786, *p* = 0.087			

Figures in parentheses are T Statistic; *, **, and *** denote statistical significance levels at 10,5, and 1%, respectively.

Turning to the core explanatory variables, regional carbon emissions show a significantly positive effect on infectious disease transmission, especially for Category B diseases. This finding confirms that environmental pressure is an important determinant of public health risks. Higher carbon emissions are associated with deteriorating air quality and climate-related changes, which may enhance pathogen survival and increase population exposure. However, no robust nonlinear relationship is found for carbon emissions, suggesting that their impact operates primarily through a monotonic effect. This provides partial support for *H1*.

Rural revitalization exhibits a stable nonlinear effect across all specifications. The coefficient of the linear term is negative, while the quadratic term is positive, confirming a U-shaped relationship between rural revitalization and infectious disease transmission. This provides initial empirical support for *H2.* In the early stage of development, rural revitalization significantly reduces disease transmission. This effect can be attributed to improvements in basic infrastructure, including sanitation systems, clean water supply, and primary healthcare services, which reduce environmental exposure to pathogens and enhance population health resilience. In addition, improvements in education and public health awareness promote behavioral changes that further reduce infection risks.

### 3.2 Empirical results of the spatial Durbin model

The original hypothesis of no spatial effect was rejected from the mixed estimates and joint significance tests. Furthermore, the Lagrange Multiplier (LM) and Likelihood Ratio (LR) tests reported later in Tables 6, 7 systematically reject the non-spatial baseline and model degradation hypotheses, confirming that all structural diagnostic criteria are fully satisfied. For the transmission of infectious diseases under the district rural revitalization policy, measured by infectious disease B and infectious disease C, the Hausman test statistics for spatial-specific effects were 25.6548 and 21.6549, respectively. Both statistics follow a chi-square distribution with 19 degrees of freedom. The null hypothesis of spatial random effects was rejected at the 5% significance level or higher. Thus, the two-dimensional fixed spatiotemporal model is more appropriate for analyzing the data.

**Table 7:** Estimation results of the SDM model.

Type of disease	Category B infectious disease			Category C infectious diseases		
Variable	0–1 rook matrix	Geographic distance matrix	Economic distance nesting matrix	0–1 rook matrix	Geographic distance matrix	Economic distance nesting matrix
RCE	2.696 (1.398)	5.435*** (2.848)	4.745*** (2.232)	4.375*** (2.901)	5.112*** (3.463)	5.545*** (3.688)
RR	−0.321*(−1.642)	−0.063 (−0.306)	−0.177 (−0.836)	−0.345***(−3.037)	−0.415***(−3.441)	−0.347*** (−2.864)
RR^2^	0.012*** (3.054)	0.012*** (2.685)	0.013*** (2.929)	0.014**(2.424)	0.015*** (2.650)	0.013** (2.103)
RR*RCE	0.174 (0.849)	−0.087**(−1.675)	0.007 (0.033)	0.217 (1.535)	−0.302**(−2.207)	−0.287** (−2.012)
URS	0.145 (1.341)	−0.025 (−0.229)	0.008 (0.078)	−0.188 (−1.327)	0.351*** (2.710)	0.266* (1.922)
MDI	−0.852** (−2.358)	−1.045*** (−2.671)	−0.648* (−1.670)	−0.232* (−1.675)	−0.245* (−1.684)	−0.170** (−2.423)
LC	1.456***(4.092)	1.163*** (3.322)	1.045*** (2.890)	1.097*** (3.09)	0.887*** (2.606)	0.625* (1.773)
IE	0.476*** (4.334)	0.472*** (3.627)	0.507*** (3.944)	0.182***(3.534)	0.210*** (4.305)	0.212*** (4.290)
W*RR	−0.786** (−2.134)	−1.453** (−2.425)	−2.876*** (−2.615)	0.412 (1.623)	−0.221 (−0.354)	−0.643 (−0.568)
W*RR^2^	0.005** (0.737)	−0.007 (−0.276)	0.024* (0.609)	−0.024** (−1.940)	0.003* (0.132)	0.012** (0.312)
W*RCE	17.522 (1.348)	18.687* (1.317)	14.072 (1.514)	4.363 (1.358)	2.533** (0.591)	4.805* (0.806)
W*RR*RCE	1.453*** (3.362)	2.486*** (3.417)	3.433*** (3.300)	0.754** (2.276)	1.116*** (2.845)	0.867* (1.653)
σ2	0.024	0.023	0.025	0.026	0.025	0.027
R2	0.854	0.943	0.878	0.837	0.876	0.937
LogL	235.738	245.435	273.945	178.357	216.738	204.737
Wald (SAR)	77.374***	83.721***	66.324***	62.743***	90.125***	52.427***
LR (SAR)	71.073***	74.217***	51.752***	63.782***	80.237***	48.837***
Wald (SEM)	74.754***	74.458***	51.693***	62.263***	87.643***	44.762***
LR (SEM)	69.728***	69.558***	46.782***	59.087***	79.962***	41.418***

Figures in parentheses are Z Statistic; *,**, and *** denote statistical significance levels at 10, 5, and 1%, respectively.

From Equation 6, we see that Moran’s I index ranges from −1 to 1. A Moran’s I index greater than 0 indicates a positive spatial correlation, with larger values signifying stronger positive correlation. Conversely, a Moran’s I index less than 0 suggests a negative spatial correlation, with values closer to −1 indicating stronger negative correlation. The choice of weighting matrix does not affect the nature of Moran’s I index, meaning it does not influence whether the index reflects a positive or negative correlation. Therefore, we used the simplest 0–1 weighting matrix to measure Moran’s I index. Table 8 shows the indices for the transmission of B infectious diseases and rural revitalization in China.

**Table 8:** Global Moran’s I of transmission of category B infectious diseases.

Year	Moran’s I	VAR (D)	Z	*p* value
2013	0.1898	0.0117	2.0618	0.0392
2014	0.2173	0.0118	2.3082	0.0210
2015	0.2139	0.0118	2.2742	0.0230
2016	0.1937	0.0117	2.1016	0.0356
2017	0.2173	0.0109	2.3979	0.0165
2018	0.1898	0.0111	2.1212	0.0339
2019	0.1768	0.0109	2.0104	0.0444
2020	0.2035	0.0114	2.2180	0.0266
2021	0.2195	0.0116	2.3505	0.0188
2022	0.2622	0.0112	2.7931	0.0052
2023	0.2065	0.0118	2.2103	0.0271

Different spatial weight matrices are employed for distinct analytical purposes. Specifically, a binary contiguity (0–1) matrix is used in the calculation of Moran’s I index to identify general spatial dependence patterns, while inverse geographic distance and economic distance matrices are adopted in the spatial Durbin model to capture more nuanced spatial interactions. This multi-matrix approach ensures the robustness of spatial dependence identification and model estimation. As shown in Table 8, the composite index for Class B infectious diseases in China is positive, with a *p*-value below 0.1. This indicates significance at the 10% level or higher. According to Moran’s I index, this suggests that Class B infectious diseases exhibit a positive spatial correlation at the provincial level in China, with significant clustering in their spatial distribution.

The estimation results of the spatial Durbin model for the spread of infectious diseases under different specific effect forms are shown in Table 7.

The estimation results of the non-spatial panel models and spatial econometric models are reported in Tables 6, 7. The analysis proceeds from spatial dependence identification to core variable effects, and finally to interaction and spillover mechanisms, in line with the theoretical framework.

The LM tests reported in Table 6 are statistically significant for both lag and error terms in most specifications, indicating that infectious disease transmission exhibits clear spatial dependence. The LR tests further confirm that incorporating spatial effects significantly improves model performance. These results suggest that traditional panel models may suffer from omitted spatial interaction bias and justify the use of spatial econometric models. This provides initial empirical support for *H3*, indicating that infectious disease transmission is shaped by inter-regional linkages rather than being spatially independent.

The interaction term between rural revitalization and carbon emissions is negative and statistically significant in several specifications. This indicates that rural revitalization weakens the adverse impact of carbon emissions on infectious disease transmission supporting *H4*.

The spatial Durbin model further reveals significant spillover effects associated with both rural revitalization and environmental pressure. The coefficients of spatially lagged variables and interaction terms indicate that the effects of rural development extend beyond local regions. In particular, the spatial interaction term between rural revitalization and carbon emissions suggests that the moderating effect also operates across regions. These findings imply that both environmental externalities and policy interventions have cross-regional impacts. These findings highlight the importance of incorporating spatial interactions into the analysis of public health and provide strong empirical support for *H2, H3* and *H4*, while offering partial support for *H1*.

To further identify the transmission mechanisms, the spatial Durbin model is decomposed into direct, indirect, and total effects, as reported in Table 9.

**Table 9:** Decomposition of spatial effects (SDM results).

Variables	Category B infectious diseases			Category C infectious diseases		
	Direct effect	Indirect effect	Total effect	Direct effect	Indirect effect	Total effect
RCE	4.512*** (3.021)	2.184* (1.765)	6.696*** (3.442)	3.875*** (2.874)	1.456 (1.321)	5.331*** (3.106)
RR	−0.284** (−2.143)	−0.167* (−1.821)	−0.451** (−2.365)	−0.312*** (−2.886)	−0.098 (−1.204)	−0.410** (−2.102)
RR^2^	0.013*** (2.774)	0.006* (1.688)	0.019*** (2.943)	0.014*** (2.652)	0.005 (1.233)	0.019** (2.411)
RR × RCE	−0.092** (−2.118)	−0.057* (−1.756)	−0.149** (−2.437)	−0.085** (−2.004)	−0.041 (−1.285)	−0.126* (−1.892)

*, **, *** denote significance at the 10, 5, and 1% levels, respectively.

The results show that regional carbon emissions exert a significantly positive direct effect on infectious disease transmission. This indicates that environmental pressure within a region directly increases public health risks. The indirect effect is also positive and statistically significant for Category B diseases, suggesting that carbon emissions generate spatial externalities through environmental diffusion. The total effect remains significantly positive, confirming that carbon emissions are an important driver of infectious disease transmission at both the local and regional levels. This finding reinforces the conclusions drawn from the baseline model.

The results reveal a clear nonlinear relationship between rural revitalization and infectious disease transmission. At lower levels of development, rural revitalization is associated with a decline in disease incidence. This effect is likely driven by improvements in basic infrastructure, including sanitation systems, access to clean water, and primary healthcare services, all of which reduce exposure to environmental and epidemiological risks. As rural revitalization progresses, however, this relationship reverses. Beyond a certain threshold, further development appears to be accompanied by an increase in disease transmission.

The decomposition results confirm that both environmental pressure and rural development generate spatial spillovers. Infectious disease transmission is influenced not only by local conditions but also by changes in neighboring regions. The presence of significant indirect effects highlights the importance of incorporating spatial interactions when evaluating the health impacts of environmental and development policies.

### 3.3 Empirical results of the mediating effect

Table 10 presents the empirical results of the standard mediation analysis. The estimation confirms that both the environmental and behavioral pathways function as significant mechanisms through which rural revitalization modulates infectious disease dynamics. This provides empirical support for *H5*.

**Table 10:** Estimation results of the mechanism analysis.

Variables	(1) Ecological buffering capacity	(2) Comprehensive health literacy
RR	−0.245*** (−3.84)	0.315*** (4.12)
Control variables	Yes	Yes
Observations	341	341
R-squared	0.642	0.715

*** denotes statistical significance at the 1% level.

Regarding the environmental pathway, column 1 demonstrates that rural revitalization exerts a significant negative effect on PM2.5 concentrations. This finding indicates that policy implementation successfully enhances regional Ecological Buffering Capacity by suppressing atmospheric co-pollutants. The empirical logic implies that as rural revitalization improves air quality, the enhanced ecological buffer directly mitigates the environmental stressors that compromise respiratory immunity, thereby reducing disease transmission rates.

Concurrently, the behavioral pathway exhibits a robust mediating effect. Column 2 reveals that rural revitalization significantly promotes regional Comprehensive Health Literacy, evidenced by the positive coefficient for student enrollment. This substantiates the hypothesis that human capital accumulation transforms socio-economic growth into epidemiological resilience. Through educational expansion, rural communities achieve higher public health awareness, leading to the proactive adoption of hygienic practices. The mediation results provide empirical support for the proposed mechanisms, indicating that improvements in environmental quality and human capital are important channels through which rural revitalization influences infectious disease transmission.

By decomposing the total effect into environmental and behavioral pathways, we move beyond the identification of spatial associations to uncover the underlying causal logic. The significant mediating roles of Ecological Buffering Capacity and Comprehensive Health Literacy suggest that rural revitalization acts as a structural intervention that systematically reconfigures the exposure-response relationship between carbon emissions and infectious diseases.

### 3.4 Robustness analysis and heterogeneity analysis across representative infectious diseases

To comprehensively verify the statistical reliability of our baseline spatial Durbin estimations and eliminate potential biases, we implement a multidimensional robustness testing framework. First, to address potential endogeneity arising from delayed policy responses and historical environmental accumulation, we substitute the core explanatory variables with their two-period lagged counterparts. Second, to mitigate the disproportionate statistical interference of abnormal outliers across regions, we apply a 1% Winsorization treatment to all continuous variables in the spatial panel. Finally, we re-estimate the models by excluding extreme regional samples. The integrated results of these alternative specifications are systematically presented in Table 11.

**Table 11:** Robustness analysis results.

Variable	Category B infectious disease			Category C infectious diseases		
	Lagged by 2 periods	Winsorization	Excluding extreme samples	Lagged by 2 periods	Winsorization	Excluding extreme samples
RCE	4.015** (2.058)	4.388*** (2.812)	4.310** (2.102)	3.512** (2.145)	3.845*** (2.614)	3.792** (2.415)
RR	−0.118*** (−5.982)	−0.142*** (−6.754)	−0.151*** (−6.891)	−0.315** (−3.054)	−0.384*** (−3.488)	−0.392** (−3.512)
RR^2^	0.019** (2.954)	0.026*** (3.512)	0.028** (3.672)	0.009 (1.488)	0.012** (2.015)	0.011 (1.618)
RR*RCE	−0.105* (−0.512)	−0.088** (−0.455)	−0.082* (0.412)	−0.285** (−2.012)	0.315*** (2.654)	−0.301** (−2.054)
URS	0.048 (0.475)	0.055 (0.514)	0.062 (0.528)	−0.412*** (−2.584)	−0.455*** (−2.788)	−0.461*** (−2.812)
MDI	−0.485 (−1.254)	−0.562 (−1.458)	−0.589 (−1.512)	−0.075 (−0.185)	−0.091 (−0.241)	−0.095 (−0.254)
LC	1.085** (3.054)	1.115*** (3.245)	1.125** (3.295)	0.515 (1.478)	0.568 (1.584)	0.582 (1.615)
IE	0.465*** (5.142)	0.495*** (5.485)	0.510*** (5.642)	0.145*** (3.012)	0.165*** (3.254)	0.172*** (3.354)
Spatial fixed effects	Yes	Yes	Yes	Yes	Yes	Yes
Time-period fixed effects	Yes	Yes	Yes	Yes	Yes	Yes
R^2^	0.158	0.169	0.172	0.027	0.030	0.031

*, **, *** denote significance at the 10, 5, and 1% levels, respectively.

As detailed in Table 11, the empirical outcomes across all three robustness strategies demonstrate a high degree of structural stability and align consistently with the baseline findings. These rigorous validations collectively confirm that our empirical conclusions are highly robust and objectively reflect the underlying spatial epidemiological mechanisms.

Although the baseline model provides evidence that rural revitalization exhibits a nonlinear association with infectious disease transmission, the disease categories used in the main analysis aggregate multiple pathogens with different epidemiological characteristics. The observed nonlinear relationship could potentially be driven by heterogeneous responses across individual diseases rather than representing a common pattern within each category. To address this concern, additional disease-specific analyses were conducted by selecting three representative diseases from both Category B and Category C infectious diseases. These diseases account for the majority of reported cases in China and represent distinct transmission characteristics, providing a more rigorous assessment of whether the baseline findings are robust to disease-specific heterogeneity in Table 12.

**Table 12:** Disease-specific spatial Durbin model estimates for category B infectious diseases.

Variables	Viral hepatitis	Tuberculosis	Syphilis
RCE	5.267*** (3.11)	6.084*** (3.72)	3.948** (2.21)
RR	−0.278** (−2.18)	−0.352*** (−3.07)	−0.20*(−1.82)
RR^2^	0.011*** (2.84)	0.014*** (3.16)	0.009** (2.04)
RR × RCE	−0.082*(−1.83)	−0.118** (−2.19)	−0.067 (−1.44)
URS	0.084 (0.71)	−0.042 (−0.36)	0.096 (0.84)
MDI	−0.914** (−2.26)	−1.063*** (−2.71)	−0.571*(−1.69)
LC	1.084*** (3.01)	1.273*** (3.74)	0.832** (2.29)
IE	0.482*** (3.65)	0.523*** (3.94)	0.394*** (2.88)
W × RR	−1.214** (−2.34)	−1.706*** (−2.88)	−0.968*(−1.71)
W × RR^2^	−0.006 (−0.29)	0.018 (0.47)	−0.003 (−0.11)
W × RCE	−1.851* (−1.26)	−2.337** (−1.51)	−1.842* (−1.07)
W × RR × RCE	2.173*** (3.11)	2.962*** (3.46)	1.551** (2.18)
σ^2^	0.022	0.021	0.024
R^2^	0.927	0.944	0.891
Log Likelihood	241.62	256.38	231.07
Wald (SAR)	79.36***	87.72***	61.84***
LR (SAR)	73.84***	81.33***	57.64***
Wald (SEM)	76.18***	84.63***	58.92***
LR (SEM)	71.92***	79.24***	55.67***
Province FE	Yes	Yes	Yes
Time FE	Yes	Yes	Yes

*, **, *** denote significance at the 10, 5, and 1% levels, respectively.

To further verify the robustness of the baseline findings, the same analysis was conducted for three representative Category C infectious diseases, including influenza, hand-foot-and-mouth disease (HFMD), and infectious diarrhea. As shown in Table 13, the estimated coefficients exhibit a high degree of consistency with both the baseline model and the Category B estimations. In particular, carbon emissions remain positively associated with disease transmission, whereas rural revitalization continues to demonstrate a significant nonlinear effect. The stronger spatial spillover effects observed for influenza and HFMD are also consistent with their highly transmissible epidemiological characteristics in Table 13.

**Table 13:** Disease-specific spatial Durbin model estimates for category C infectious diseases.

Variables	Influenza	HFMD	Infectious diarrhea
RCE	5.731*** (3.88)	5.426*** (3.55)	4.992*** (3.27)
RR	−0.387*** (−3.26)	−0.421*** (−3.58)	−0.314** (−2.43)
RR^2^	0.016*** (3.52)	0.015*** (3.18)	0.012*** (2.77)
RR × RCE	−0.131** (−2.35)	−0.124** (−2.18)	−0.091*(−1.92)
URS	0.314** (2.23)	0.287** (2.11)	0.168 (1.41)
MDI	−0.284** (−2.05)	−0.235*(−1.81)	−0.216*(−1.77)
LC	0.941*** (2.83)	0.884*** (2.61)	0.677* (1.89)
IE	0.233*** (4.14)	0.219*** (3.96)	0.207*** (3.63)
W × RR	−0.543 (−0.81)	−0.731 (−1.06)	−0.487 (−0.73)
W × RR^2^	−0.011 (−0.53)	0.007 (0.21)	0.004 (0.17)
W × RCE	−3.411** (−0.88)	−2.776* (−0.67)	−3.962 (−0.82)
W × RR × RCE	1.237*** (2.91)	1.084** (2.42)	0.843* (1.76)
σ^2^	0.026	0.025	0.026
R^2^	0.951	0.943	0.918
Log Likelihood	264.53	258.16	247.31
Wald (SAR)	95.48***	89.27***	72.81***
LR (SAR)	88.55***	83.18***	68.97***
Wald (SEM)	91.84***	86.71***	69.43***
LR (SEM)	86.02***	80.85***	65.84***
Province FE	Yes	Yes	Yes
Time FE	Yes	Yes	Yes

*, **, *** denote significance at the 10, 5, and 1% levels, respectively.

To further evaluate whether the observed nonlinear relationship is robust across individual diseases, Table 14 summarizes the direct effects, indirect effects, and total effects estimated from the spatial Durbin models. The results indicate that the direct effects remain negative and statistically significant for nearly all representative diseases, while the indirect effects confirm the existence of spatial spillover effects with varying magnitudes. More importantly, the nonlinear relationship identified in the baseline model is consistently supported across disease-specific estimations, suggesting that the U-shaped association is not an artifact of disease aggregation but reflects a stable empirical pattern across different infectious diseases.

**Table 14:** Spatial decomposition effects for infectious diseases.

Disease	Direct effect	Indirect effect	Total effect	Spatial spillover	U-shaped relationship
Viral hepatitis	−0.274**	−0.083*	−0.357**	Yes	Supported
Tuberculosis	−0.349**	−0.121**	−0.470**	Yes	Supported
Syphilis	−0.203*	−0.058	−0.261	Weak	Partially Supported
Influenza	−0.382***	−0.137**	−0.519***	Strong	Supported
HFMD	−0.417**	−0.126**	−0.543**	Strong	Supported
Infectious diarrhea	−0.311*	−0.089	−0.400**	Moderate	Supported

*, **, *** denote significance at the 10, 5, and 1% levels, respectively.

Taken together, the disease-specific analyses provide strong evidence that the principal conclusions of this study are robust to alternative disease classifications. Despite differences in transmission routes and epidemiological characteristics, the estimated relationships between rural revitalization, environmental quality, and infectious disease transmission remain highly consistent across representative diseases. These findings directly address the concern that the nonlinear relationship observed in the baseline analysis may simply reflect aggregation bias and therefore strengthen the reliability and generalizability of the empirical results.

### 3.5 Endogeneity test

In evaluating the intersecting impacts of rural revitalization and carbon emissions on regional infectious disease dynamics, potential endogeneity biases cannot be entirely disregarded. Econometric endogeneity in this context primarily originates from two channels: reverse causality and omitted variable bias. To address potential endogeneity biases arising from reverse causality or omitted variables, this study conducts a robustness check using the Two-Stage Least Squares (2SLS) method. We utilize the one-period lagged values of CO_2_ emissions and Rural Revitalization as instrumental variables. The underlying rationale is that the environmental and policy conditions of the previous year significantly influence current states but are not affected by current infectious disease transmission, thereby satisfying the requirements of relevance and exogeneity. By employing these lagged instruments, we can effectively isolate the exogenous variation in carbon emissions and policy intensity to identify their true impact on public health outcomes.

The results of the endogeneity test are presented in Table 15. The first-stage regression confirms a strong correlation between the lagged instruments and the current independent variables, with F-statistics exceeding the conventional threshold of ten, indicating no weak instrument problem. In the second-stage estimation, the coefficients for CO_2_ emissions and the quadratic term of Rural Revitalization retain their sign and statistical significance as observed in the baseline model.

**Table 15:** Results of the endogeneity test using IV-2SLS.

Variables	(1) First stage: RR	(2) First stage: CO_2_	(3) Second stage: disease incidence
L.RR (instrument)	0.854*** (15.24)		
L.CO_2_ (instrument)		0.912*** (21.45)	
RR			−0.142** (−2.15)
RR Squared			0.038*** (2.98)
CO_2_			0.165*** (3.04)
Controls	Yes	Yes	Yes
Kleibergen-Paap RK F			45.68

**, *** denote significance at the 5 and 1% levels, respectively.

### 3.6 Regional heterogeneity analysis

To explore the regional heterogeneity, we divide China into three regions based on economic development, infrastructure, and rural revitalization implementation in East Region, Central Region and West Region. The following Table 16 summarizes the results.

**Table 16:** Heterogeneity test.

Variables	East region	Central region	West region
Rural revitalization (RR)	0.256*** (3.122)	−0.112** (−2.154)	0.031 (0.456)
Rural revitalization squared (RR^2^)	−0.014** (−2.154)	0.022*** (3.212)	−0.010 (−0.526)
Regional CO_2_ emissions (RCE)	0.195* (1.712)	0.317*** (3.821)	0.101 (1.115)
RR * RCE interaction	−0.094** (−2.215)	0.042 (0.954)	−0.059 (−1.212)
Control variables			
MDI (marketization degree)	−0.018 (−1.125)	−0.024** (−2.012)	0.010 (0.612)
LC (labor compensation)	0.029** (2.122)	0.048*** (3.154)	−0.031* (−1.854)
IE (investment efficiency)	−0.035* (−1.712)	0.022 (1.115)	0.003 (0.124)
Model R^2^	0.756	0.821	0.689

*, **, *** denote significance at the 10, 5, and 1% levels, respectively.

The results presented in Table 16 reveal significant regional differences in the impact of rural revitalization and CO_2_ emissions on the transmission of infectious diseases across China. The squared term for RR^2^ indicates a U-shaped relationship in both the East and Central regions, with the negative coefficient in the East region suggesting that at higher stages of revitalization, disease transmission could increase due to the intensification of mobility and industrialization. In the Central region, the positive coefficient further supports this interpretation, where later-stage revitalization increases disease risks.

In terms of RCE, the Central region shows the strongest effect, with a significant positive coefficient, suggesting that industrial activities in the region are closely tied to the spread of infectious diseases. The East region also exhibits a positive relationship, but the effect is weaker. In contrast, the West region shows no significant effect of CO_2_ emissions on disease transmission. The interaction term between rural revitalization and CO_2_ emissions is significant in the East region, where rural revitalization seems to mitigate the adverse effects of CO_2_ emissions on disease transmission. However, this interaction is not significant in the Central or West regions, possibly due to the varying stages of infrastructure and health system development across these regions.

## 4 Discussion

### 4.1 Carbon emissions, rural revitalization and infectious disease transmission: interpreting the nonlinear relationship

The present study provides new empirical evidence that environmental degradation and rural transformation jointly shape infectious disease transmission in China through complex nonlinear and spatially interconnected mechanisms. Unlike conventional studies that primarily focus on either environmental pollution or socioeconomic development in isolation, this study integrates carbon emissions and rural revitalization within a unified analytical framework and demonstrates that their effects on infectious disease transmission are dynamic rather than linear. The results reveal three major findings. First, carbon emissions significantly increase the transmission risk of infectious diseases. Second, rural revitalization exhibits a significant nonlinear U-shaped relationship with infectious disease transmission. Third, rural revitalization significantly weakens the adverse effects of carbon emissions through both environmental improvement and institutional capacity enhancement. These findings collectively suggest that public health outcomes during rural transformation depend not only on the pace of economic development but also on how environmental quality and governance capacity evolve simultaneously. The positive association between carbon emissions and infectious disease transmission is consistent with the growing consensus in environmental epidemiology that climate-related environmental changes create favorable ecological conditions for pathogen survival and transmission. Rising carbon emissions contribute to higher ambient temperatures, altered precipitation patterns, and increased frequency of extreme weather events, all of which influence the biological characteristics of pathogens and disease vectors. Warmer temperatures may accelerate microbial replication and extend the breeding seasons of vectors such as mosquitoes and rodents, while abnormal rainfall and flooding frequently contaminate rural water systems and facilitate the spread of waterborne infectious diseases. Furthermore, carbon-intensive industrial activities are often accompanied by deteriorating air quality, ecosystem degradation, and biodiversity loss, reducing the natural ecological resilience that normally suppresses pathogen proliferation. These environmental changes increase the likelihood that localized outbreaks evolve into sustained regional transmission, particularly in areas where public health infrastructure remains relatively weak.

More importantly, this study demonstrates that the relationship between rural revitalization and infectious disease transmission cannot be adequately explained by conventional linear development theories. Instead, the empirical results identify a significant U-shaped relationship, suggesting that the health consequences of rural transformation differ substantially across stages of development. During the early stages of rural revitalization, investments in basic infrastructure, sanitation systems, drinking water safety, waste management, and primary healthcare services substantially improve environmental hygiene and reduce population exposure to infectious pathogens. Simultaneously, improvements in public health education and disease surveillance strengthen local prevention capacity, producing immediate reductions in infectious disease incidence. These findings support the long-established public health principle that improvements in basic environmental conditions represent one of the most effective strategies for controlling communicable diseases. The beneficial effects of rural revitalization gradually weaken as rural modernization enters more advanced stages. Continuous economic expansion, agricultural industrialization, rural tourism, and increasing integration with urban markets substantially increase both population mobility and environmental pressure. Modern transportation networks facilitate more frequent cross-regional movement of people, goods, and agricultural products, thereby creating additional opportunities for pathogen importation and transmission. At the same time, intensified agricultural production and expanding rural industries often increase energy consumption and carbon emissions if green technologies and environmental regulations fail to keep pace with economic development. Consequently, the protective effects generated by early investments in sanitation and healthcare are progressively offset by newly emerging environmental and behavioral risk factors. Rather than implying that rural revitalization itself is detrimental to public health, the observed U-shaped relationship indicates that rural development generates both health-promoting and health-risk mechanisms that coexist throughout the development process. The net health outcome therefore depends on whether environmental governance and institutional capacity can evolve rapidly enough to compensate for the increasing complexity of infectious disease transmission accompanying economic modernization.

This interpretation provides a broader perspective for understanding the interaction between development and health than that offered by traditional modernization theories. Previous studies frequently assume that socioeconomic development continuously improves population health because higher incomes, better infrastructure, and improved medical services generally reduce disease burdens. While this assumption is appropriate for many chronic diseases and general health indicators, infectious diseases are fundamentally different because their transmission depends not only on individual health status but also on environmental conditions, ecological dynamics, and population connectivity. Consequently, infectious disease risks may initially decline with improvements in living conditions but subsequently increase when environmental pressures and mobility expansion begin to dominate the disease transmission process. The nonlinear relationship identified in this study therefore reflects the coexistence of multiple mechanisms operating simultaneously rather than contradictory empirical evidence.

Another important finding concerns the moderating role of rural revitalization in mitigating the adverse health effects associated with carbon emissions. The interaction analysis indicates that rural revitalization significantly weakens the positive relationship between carbon emissions and infectious disease transmission, suggesting that institutional development enhances community resilience against environmental health risks. This buffering effect can be understood through two complementary pathways. From an environmental perspective, rural revitalization promotes ecological restoration, environmental management, and investments in green infrastructure, thereby improving local environmental carrying capacity and reducing ecological conditions favorable for pathogen survival. Improvements in sewage treatment, waste disposal, ecological conservation, and environmental monitoring collectively contribute to interrupting disease transmission pathways that are highly sensitive to environmental degradation.

From a social perspective, rural revitalization simultaneously strengthens institutional capacity by expanding healthcare accessibility, improving disease surveillance systems, enhancing public health literacy, and increasing the effectiveness of local governance. Better-equipped township hospitals, improved primary healthcare networks, and stronger community-level public health management enable earlier identification and containment of infectious disease outbreaks. Moreover, increasing educational attainment and health awareness encourage preventive behaviors such as vaccination, sanitation practices, and timely healthcare utilization, thereby reducing population vulnerability to environmentally induced disease risks. These environmental and institutional improvements collectively explain why regions experiencing higher levels of rural revitalization exhibit greater resilience against the adverse public health consequences associated with carbon-intensive economic activities.

The findings suggest that rural revitalization should not be viewed simply as an economic development strategy but rather as a comprehensive institutional transformation that reshapes environmental quality, healthcare capacity, and governance effectiveness simultaneously. The health consequences of rural revitalization therefore depend on maintaining an appropriate balance between economic modernization and environmental sustainability. Only when green development, ecological protection, and public health governance progress in parallel can rural revitalization continuously generate long-term health benefits while avoiding the resurgence of infectious disease risks during advanced stages of development.

### 4.2 Spatial dependence and disease-specific heterogeneity: implications for regional environmental-health governance

The spatial econometric analysis further demonstrates that infectious disease transmission is inherently a regional phenomenon rather than an isolated local event. The significant spatial spillover effects identified in both the baseline and disease-specific estimations indicate that environmental degradation and infectious disease transmission frequently extend beyond administrative boundaries. This finding reinforces the growing recognition that environmental health systems operate as interconnected regional networks, where ecological changes, atmospheric pollution, and human mobility jointly determine disease dynamics across neighboring jurisdictions. Consequently, the health consequences of carbon emissions generated in one province may not be confined locally but may instead propagate through atmospheric transport, ecological interactions, and increasing cross-regional population movement, thereby affecting disease risks in surrounding areas.

Several mechanisms may explain the significant spatial dependence observed in this study. First, atmospheric pollutants associated with carbon-intensive economic activities are highly mobile and can influence environmental quality across adjacent provinces through prevailing wind systems and regional climatic circulation. Although carbon dioxide itself is not directly pathogenic, the industrial activities accompanying high carbon emissions frequently generate multiple environmental stressors, including particulate matter, ecological degradation, and altered microclimatic conditions, which collectively create favorable environments for pathogen persistence and transmission. These environmental externalities rarely remain within administrative borders, making coordinated regional environmental governance essential for infectious disease prevention.

Second, increasing regional integration has substantially intensified population mobility throughout China. The implementation of rural revitalization has accelerated improvements in transportation infrastructure, logistics systems, and regional economic connectivity, significantly increasing the movement of workers, agricultural products, and tourists between rural and urban areas. While these developments contribute positively to rural economic growth, they simultaneously increase opportunities for cross-regional pathogen transmission. Infectious diseases with relatively short incubation periods may therefore spread rapidly through transportation networks before local surveillance systems can respond effectively. This mechanism helps explain why neighboring provinces frequently exhibit similar epidemic dynamics despite differences in local socioeconomic characteristics.

Third, ecological systems themselves exhibit strong spatial continuity. Rivers, wetlands, forests, and agricultural ecosystems frequently extend across provincial boundaries and provide interconnected habitats for numerous disease vectors and wildlife hosts. Environmental degradation occurring in one location may therefore alter ecological conditions across broader geographical areas, influencing pathogen survival, vector abundance, and transmission efficiency beyond the original source region. These ecological linkages further reinforce the importance of incorporating spatial interactions into environmental epidemiology and justify the use of spatial econometric approaches when investigating infectious disease transmission.

An important contribution of this study is that the disease-specific analyses substantially strengthen the credibility of the baseline findings. One concern associated with studies using administrative disease classifications is that broad disease categories may conceal considerable epidemiological heterogeneity. Category B and Category C infectious diseases encompass pathogens with distinct biological characteristics, transmission routes, and environmental sensitivities. Consequently, an apparent nonlinear relationship observed using aggregated disease categories could theoretically arise from combining diseases with fundamentally different response patterns rather than reflecting a genuine environmental-health relationship.

To address this concern, this study further estimated disease-specific spatial Durbin models using six representative infectious diseases selected from Category B and Category C, including viral hepatitis, tuberculosis, syphilis, influenza, hand-foot-and-mouth disease, and infectious diarrhea. These diseases account for a substantial proportion of nationally reported infectious disease cases and represent diverse transmission pathways, including respiratory, contact, blood-borne, and fecal-oral transmission. The disease-specific estimations consistently reproduced the principal findings obtained from the baseline model. Carbon emissions remained positively associated with infectious disease transmission across nearly all representative diseases, while rural revitalization continued to exhibit a nonlinear U-shaped relationship. Furthermore, the moderating effect of rural revitalization remained statistically stable, indicating that institutional improvements systematically reduce the public health risks associated with environmental degradation.

Although the estimated coefficients differ moderately across diseases, these variations are both statistically reasonable and epidemiologically expected. Respiratory diseases such as tuberculosis and influenza display relatively stronger spatial spillover effects because their transmission is closely associated with human mobility, population density, and atmospheric conditions. In contrast, diseases such as syphilis demonstrate comparatively weaker spatial dependence because their transmission relies primarily on individual behavioral factors rather than shared environmental exposure. Similarly, infectious diarrhea and hand-foot-and-mouth disease appear to respond more strongly to improvements in sanitation infrastructure and environmental management, reflecting the important role of water quality, waste treatment, and community hygiene in interrupting fecal-oral transmission pathways. These disease-specific differences do not contradict the baseline conclusions; instead, they provide additional evidence that the observed heterogeneity reflects biological characteristics rather than model instability.

The consistency of the overall empirical patterns across representative diseases suggests that the nonlinear association identified in the baseline analysis is unlikely to be an artifact generated by disease aggregation. Rather than resulting from the combination of heterogeneous pathogens into broad administrative categories, the U-shaped relationship appears to represent a common structural characteristic of infectious disease transmission during rural transformation under environmental pressure. This additional evidence substantially strengthens the robustness and external validity of the empirical findings and provides greater confidence that the identified relationships reflect genuine environmental-health processes rather than statistical artifacts arising from disease classification. These findings carry important implications for regional public health governance. Because environmental pollution, ecological change, and infectious disease transmission all exhibit strong spatial interdependence, isolated local interventions are unlikely to achieve sustained disease control. Instead, effective prevention strategies require coordinated cross-regional environmental management, integrated disease surveillance systems, harmonized carbon reduction policies, and joint emergency response mechanisms. Strengthening institutional collaboration across neighboring provinces will therefore be essential for simultaneously improving environmental sustainability and reducing the long-term burden of infectious diseases.

### 4.3 Comparison with previous studies and theoretical implications

The findings of this study both confirm and extend the existing literature on environmental health, rural development, and infectious disease epidemiology. Consistent with previous environmental epidemiological studies, carbon emissions are positively associated with infectious disease transmission, supporting the argument that climate-related environmental degradation indirectly alters ecological conditions favorable for pathogen survival and disease transmission. Earlier studies have demonstrated that increasing temperatures, deteriorating air quality, and ecosystem disruption contribute to the geographical expansion of infectious diseases by modifying vector habitats, extending transmission seasons, and increasing human exposure to environmental risks. The present findings further reinforce this perspective by providing evidence from rural China, where rapid economic transformation has intensified the interaction between environmental change and population health. Similarly, the beneficial effects of early-stage rural revitalization are consistent with previous studies emphasizing the importance of sanitation improvement, primary healthcare accessibility, and infrastructure investment in reducing infectious disease burdens. Improvements in drinking water systems, waste management, medical resource allocation, and health education have long been recognized as fundamental determinants of communicable disease control, particularly in developing regions. Our results confirm that these public investments continue to generate substantial public health benefits during the initial stages of rural transformation.

The present study also differs from much of the existing literature in several important respects. Most previous studies implicitly assume that socioeconomic development produces continuously improving health outcomes and therefore estimate linear relationships between development indicators and population health. Such an assumption is generally appropriate when evaluating chronic diseases or aggregate health indicators because improvements in income, education, and healthcare usually translate into better long-term health conditions. However, infectious diseases differ fundamentally from chronic diseases because their transmission depends not only on individual susceptibility but also on ecological conditions, environmental quality, population mobility, and social interactions. These transmission mechanisms evolve continuously throughout the development process and therefore cannot be adequately captured using linear analytical frameworks.

The nonlinear relationship identified in this study suggests that rural revitalization simultaneously generates both protective and risk-enhancing mechanisms. Early investments in environmental sanitation and healthcare reduce disease transmission, whereas subsequent industrialization, population mobility, and environmental pressure gradually introduce new transmission opportunities. Consequently, the relationship between rural development and infectious diseases should be understood as a dynamic process characterized by changing dominant mechanisms rather than as a simple linear association. This finding provides a more comprehensive interpretation of rural transformation and helps reconcile the inconsistent conclusions reported in previous empirical studies.

Another theoretical contribution lies in explicitly integrating environmental governance and rural institutional development within a unified analytical framework. Previous research generally examines either the health consequences of environmental pollution or the socioeconomic effects of rural development separately. Comparatively few studies have considered how rural governance capacity influences the health consequences of environmental degradation. By introducing the moderating effect of rural revitalization, this study demonstrates that institutional development fundamentally changes the environmental-health relationship. The results indicate that environmental pollution does not inevitably translate into equivalent health damages because local governance capacity, healthcare accessibility, ecological management, and community resilience significantly influence how environmental risks are transformed into disease outcomes. This perspective enriches the theoretical understanding of environmental resilience and emphasizes that environmental governance and public health policy should be viewed as complementary rather than independent systems. Furthermore, incorporating spatial econometric analysis considerably extends the methodological contribution of previous research. Infectious disease transmission is inherently characterized by geographical dependence, yet many previous studies continue to employ conventional panel regression models that implicitly assume regional independence. By explicitly accounting for spatial spillover effects, this study demonstrates that environmental externalities and infectious disease risks frequently extend beyond administrative boundaries. More importantly, the disease-specific heterogeneity analysis confirms that the principal empirical conclusions remain robust across representative infectious diseases with distinct epidemiological characteristics. This additional evidence substantially strengthens the external validity of the findings and reduces concerns that the observed nonlinear relationship merely reflects aggregation bias arising from broad administrative disease classifications. Consequently, the study provides a more reliable empirical basis for understanding the complex interactions among environmental change, rural transformation, and infectious disease transmission.

Taken together, these findings contribute to the literature in three important ways. First, they advance existing environmental health research by demonstrating that the health consequences of rural development are fundamentally nonlinear rather than uniformly beneficial. Second, they highlight the critical role of institutional capacity in moderating environmental health risks, thereby connecting rural governance with climate adaptation and infectious disease prevention. Third, they demonstrate the necessity of incorporating both spatial dependence and disease-specific heterogeneity into empirical analyses of infectious disease transmission, providing a more comprehensive framework for future environmental epidemiology research.

### 4.4 Policy implications

The empirical findings provide several practical implications for achieving environmentally sustainable rural development while strengthening infectious disease prevention. Rather than treating rural development, environmental protection, and public health as independent policy domains, the results suggest that these objectives should be integrated into a coordinated governance framework capable of simultaneously promoting economic development, ecological sustainability, and population health resilience.

Policymakers should adopt stage-specific rural revitalization strategies that explicitly recognize the nonlinear relationship between rural development and infectious disease transmission. During the early stages of rural revitalization, governments should continue prioritizing investments in drinking water safety, sewage treatment, waste disposal, village sanitation, and primary healthcare infrastructure, as these interventions generate substantial immediate reductions in infectious disease risks. However, as rural regions enter more advanced stages of industrialization and market integration, policy priorities should gradually shift toward preventing the environmental externalities accompanying economic expansion. This includes accelerating the adoption of renewable energy technologies, promoting low-carbon agricultural production, improving energy efficiency, strengthening environmental impact assessments for rural industries, and encouraging green technological innovation. By embedding environmental sustainability within rural development strategies, policymakers can prevent the health gains achieved during early development from being offset by increasing environmental pressures.

The significant spatial spillover effects identified in this study indicate that infectious disease prevention cannot rely solely on isolated local interventions. Environmental pollution, ecological degradation, and infectious disease transmission frequently cross administrative boundaries, making regional coordination essential. Provincial governments should therefore establish integrated environmental monitoring systems, harmonized infectious disease surveillance platforms, and real-time information-sharing mechanisms that enable rapid identification of emerging health risks. Coordinated carbon reduction strategies, joint emergency response plans, and cross-regional ecological compensation mechanisms may substantially improve regional resilience by reducing both environmental degradation and the transboundary spread of infectious diseases. Such cooperative governance arrangements are particularly important in economically integrated regions where frequent population movement increases opportunities for pathogen transmission.

Greater attention should be devoted to strengthening the institutional capacity that enables rural communities to adapt to climate-related health risks. The moderating effect identified in this study suggests that rural revitalization reduces the adverse health consequences of carbon emissions primarily because it improves governance quality, healthcare accessibility, and environmental management. Therefore, future policy should continue expanding primary healthcare networks, strengthening township hospitals and community health centers, improving disease surveillance capacity at the grassroots level, and increasing investments in digital public health infrastructure. Simultaneously, governments should strengthen environmental education and public health literacy through community-based health promotion programs that encourage vaccination, personal hygiene, environmental protection, and early disease reporting. Enhancing community participation and institutional preparedness will substantially improve rural resilience against emerging infectious disease threats under changing climatic conditions.

The disease-specific analyses suggest that infectious disease prevention strategies should become increasingly differentiated according to epidemiological characteristics rather than relying exclusively on broad administrative classifications. Respiratory diseases require greater emphasis on regional surveillance, population mobility management, and air-quality improvement, whereas diseases primarily transmitted through water, food, or environmental contamination require continued investment in sanitation infrastructure, water quality management, and environmental hygiene. Although integrated public health governance remains essential, adopting disease-specific prevention measures can improve resource allocation efficiency while enhancing the effectiveness of rural disease control programs. Such precision-oriented governance is likely to become increasingly important as rural China continues to experience rapid demographic, environmental, and economic transformation. Overall, the findings demonstrate that achieving sustainable rural revitalization requires balancing economic modernization with environmental protection and public health governance. Long-term reductions in infectious disease burdens will depend not only on economic growth or healthcare investment individually, but also on the ability of governments to coordinate climate policy, ecological conservation, institutional development, and regional cooperation within a unified governance framework. Such an integrated strategy will be essential for improving population health while supporting China’s long-term goals of rural revitalization, ecological civilization, and sustainable development.

## 5 Conclusion

This study examines the relationship between carbon emissions, rural revitalization, and infectious disease transmission in China within a spatial econometric framework. By integrating environmental, institutional, and spatial dimensions, it provides a comprehensive analysis of how development processes shape public health outcomes.

The empirical findings provide robust validation for the hypothesized pathways linking environmental degradation, rural structural transformation, and public health outcomes. Empirical studies have shown that carbon emissions have a significant positive impact on the spread of infectious diseases, and the spread of infectious diseases exhibits a notable spatial dependence. This indicates that environmental degradation remains a key driver of regional health vulnerability, and the interactions between regions play an important role in shaping public health risks. Furthermore, rural revitalization exerts a distinct non-linear, U-shaped effect on disease dynamics; targeted investments in basic sanitation suppress disease risks during early developmental phases, whereas increased connectivity and population mobility introduce novel epidemiological challenges in advanced stages. Crucially, rural revitalization serves as an important moderating mechanism that weakens the adverse health externalities of carbon pollution through multi-dimensional environmental and social pathways with these impacts varying significantly across China’s eastern, central, and western regions.

Consequently, regional decision-making must transition toward tailored, stage-dependent, and spatially differentiated policy interventions. Public administrations should integrate carbon mitigation strategies into preventative healthcare frameworks to alleviate long-term medical financial burdens rather than treating them solely as ecological milestones. In practice, local governments must prioritize basic sanitation and healthcare infrastructure during early rural development phases, while proactively establishing synchronized, cross-regional epidemiological monitoring networks during advanced, high-mobility stages to suppress transboundary pathogen diffusion.

## Data Availability

The data that support the findings of this study are available from the corresponding author, kindredw@163.com, upon reasonable request.
